# Heart rate lowering treatment leads to a reduction in vulnerable plaque features in atherosclerotic rabbits

**DOI:** 10.1371/journal.pone.0179024

**Published:** 2017-06-22

**Authors:** Raf H. M. van Hoof, Evelien Hermeling, Judith C. Sluimer, Julie Salzmann, Arnold P. G. Hoeks, Jérôme Roussel, Mat J. A. P. Daemen, Harry Struijker-Boudier, Joachim E. Wildberger, Sylvia Heeneman, M. Eline Kooi

**Affiliations:** 1 Department of Radiology, Maastricht University Medical Center, Maastricht, The Netherlands; 2 CARIM School for Cardiovascular Diseases, Maastricht University, Maastricht, The Netherlands; 3 Department of Pathology, Maastricht University Medical Center, Maastricht, The Netherlands; 4 Institut de Recherches Internationals Servier, Suresnes, France; 5 Department of Biomedical Engineering, Maastricht University, Maastricht, The Netherlands; 6 Department of Pathology, Academic Medical Center, Amsterdam, Netherlands; 7 Department of Pharmacology, Maastricht University, Maastricht, The Netherlands; Universitatsklinikum Freiburg, GERMANY

## Abstract

**Objective:**

To investigate the effect of a heart rate (HR) lowering agent (Ivabradine) on features of atherosclerotic plaque vulnerability with magnetic resonance imaging (MRI), ultrasound imaging, and histology.

**Approach and results:**

Atherosclerosis was induced in the abdominal aorta of 19 rabbits. Nine rabbits were treated with Ivabradine (17 mg/kg/day) during the entire study period. At week 14, imaging was performed. Plaque size was quantified on contrast-enhanced T1-weighted MR images. Microvascular flow, density, and permeability was studied with dynamic contrast-enhanced MRI. Plaque biomechanics was studied by measuring the aortic distension with ultrasound. After, animals were sacrificed and histology was performed.

HR was reduced by 16% (p = 0.026) in Ivabradine-treated animals. No differences in absolute and relative vessel wall beat-to-beat distension were found, but due to the reduction in HR, the frequency of the biomechanical load on the plaque was reduced. Plaque size (MR and histology) was similar between groups. Although microvessel density (histology) was similar between groups, AUC and K^trans^, indicative for plaque microvasculature flow, density, and permeability, were decreased by 24% (p = 0.029) and 32% (p = 0.037), respectively. Macrophage content (relative RAM11 positive area) was reduced by 44% (p<0.001) on histology in Ivabradine-treated animals.

**Conclusions:**

HR lowering treatment with Ivabradine in an atherosclerotic rabbit model is associated with a reduction in vulnerable plaque features. The current study suggests that HR reduction may be beneficial for inducing or maintaining a more stable plaque phenotype.

## Introduction

### Increased heart rate and cardiovascular disease

Cardiovascular diseases continue to be the major cause of morbidity and mortality in the world. Large epidemiologic studies have shown that an elevated resting heart rate (HR) is an independent predictor of life expectancy, in subjects with and without diagnosed cardiovascular disease [1–3]. Rupture of a vulnerable atherosclerotic plaque is an important underlying cause of cardiovascular events such as myocardial infarction and stroke. Experimental animal studies have demonstrated a link between HR and the burden of both coronary and carotid atherosclerosis [4–10]. An increased HR may play a role in the progression of atherosclerosis due to the increased frequency of the biomechanical load imposed on the vessel wall (i.e. repetitive wall strain). Every heart beat, an atherosclerotic plaque is exposed to the arterial pressure wave, inducing wall stress, leading to repetitive plaque deformation (i.e. strain). Plaque strain may result in minor tissue damage, which can accumulate in time (crack propagation). This is also referred to as the fatigue hypothesis [11, 12]. Hence, intensity of the biomechanical load on the plaque depends on heart rate as well as magnitude of repetitive wall stress. A high biomechanical load could lead to rupture of plaque microvessels, cap fissures, and ultimately, cap rupture. Ruptured microvessels or fissures may then provide a point of entry for inflammatory cells and erythrocytes [13] into the plaque, leading to further plaque destabilization [14].

### In vivo imaging of atherosclerosis and plaque strain

Atherosclerosis can be visualized non-invasively using several imaging techniques. Ultrasound is frequently been used to assess the degree of stenosis of a blood vessel [15]. In addition, ultrasound can be used to assess biomechanical properties of the vessel wall. The local wall strain coefficient is inversely related to the distensibility coefficient [16], i.e. the relative change in lumen diameter for a given pressure change. The distensibility coefficient in the carotid artery of humans is positively associated with the incidence of cardiovascular events [17]. The (change in) lumen diameter can be measured over the cardiac cycle by phase tracking of raw radiofrequency ultrasound signals, providing high accuracy and precision [18]. Magnetic Resonance Imaging (MRI) has emerged as a promising technique to visualize both plaque size and features of plaque vulnerability in vivo [19]. Dynamic contrast-enhanced MRI (DCE-MRI) has developed into a tool for assessment of the atherosclerotic plaque microvasculature [20–30], which is thought to be a hallmark of plaque vulnerability [31]. It has been shown that both semi-quantitative [20] and quantitative [25–27] DCE-MRI parameters correlate with the amount of microvessels in the plaque. Previous research [22–24] has shown that anti-inflammatory treatment is associated with changes in DCE-MRI derived parameters.

### Selective heart rate reduction

Ivabradine (Servier, Suresnes, France) reduces the HR by specific and selective inhibition of the sinus node [32] with a high specificity [33], but does not affect cardiac contractility or blood pressure (BP) [33, 34]. Thus, Ivabradine treatment provides a model to study the effects of HR reduction on plaque size and vulnerability. A pre-clinical histopathological study has shown that Ivabradine treatment reduced atherosclerosis in ApoE^-/-^ mice [35]. However, no in vivo imaging was performed. In addition dynamic processes, like (leaky) plaque microvasculature and strain were not investigated.

### Aim of the study

In the present study, the effect of a reduced HR by Ivabradine treatment on atherosclerotic rabbit plaques was studied. We aimed to examine plaque burden and (leaky) plaque microvasculature with MRI. Local absolute and relative distension of the vessel wall was assessed using high-frame rate ultrasound. Features of plaque vulnerability (i.e. relative lipid-rich necrotic core size, microvessel density, and macrophage content) were quantified using histology.

## Material and methods

### Experimental methods

#### Animal model

**Fig 1:**
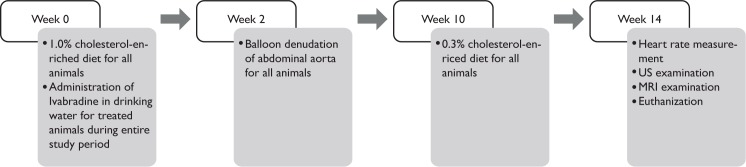
The experimental design. Two weeks after initiation of 1.0% cholesterol-enriched diet, the animals receive a balloon injury. At week 10, they are switched from 1.0% to 0.3% cholesterol-enriched diet. Fourteen weeks after diet initiation the animals undergo measurement of the HR and US and MR imaging before euthanisation.

All experiments were approved by the Maastricht University Animal Experiments Committee. Atherosclerosis was induced in nineteen 12 weeks old male New Zealand white rabbits (Charles River, Romans, France) using a modified version of a previous protocol [36]. The experimental design is presented in Fig 1. In short, animals were given a 1.0% cholesterol–enriched diet for 10 weeks, followed by a 0.3% cholesterol-enriched diet for 4 weeks. During the study, animals were given normal drinking water (n = 10) or drinking water with Ivabradine (n = 9) (17 mg/kg/day). The dosage was adjusted according to animal weight and water consumption on a weekly basis.

After two weeks, animals underwent a balloon denudation of the abdominal aorta. During this procedure, rabbits were anesthetized using ketamine (15 mg/kg) and medetomidine (0.25 mg/kg) and intubated with an endotracheal tube. Active oxygen ventilation with 1–2% isoflurane inhalation gas was performed during the rest of the experiment. The left carotid artery was exposed, and a 4-F catheter introducer sheath was inserted (Avanti Plus; Cordis Europe, Brussels, Belgium) until the aortic arch was reached. Next, a guidewire (Radiofocus Guidewire M; Terumo Europe, Brussels, Belgium) was inserted through the sheath until in the descending aorta was reached. A 3-F Fogarty embolectomy catheter (Edwards Lifesciences, Irvine, California) was then inserted, and was positioned 1 cm proximally to the aortic bifurcation. From this point, the catheter was inflated and withdrawn over 5 cm for three times. After removal of the devices, the carotid artery was ligated and the wound was sutured. Animals were hourly monitored during post-surgery recovery by monitoring heart rate, oxygen saturation, and breathing frequency.

After 14 weeks, animals underwent US and MR imaging of the abdominal aorta under general anesthesia as described before. Before anesthesia, conscious heart rate was measured using pulse oximetry. During ultrasound imaging, systolic and diastolic BP were measured in the auricular artery. After the imaging experiments, animals were sacrificed by an overdose sodium pentobarbital.

#### US protocol

The distension, i.e. the maximal change in aortic diameter over the cardiac cycle, was assessed using a PICUS ultrasound scanner (Esaote Europe, Maastricht, Netherlands) with a 7.5MHz linear array probe. The system was operating in multiple M-mode, producing 28 M-mode lines at a frame rate of 380 Hz [18], covering a segment of the abdominal aorta of 26.5 mm. The radiofrequency-data were sampled at 33.3 MHz.

#### MRI protocol

MRI experiments were performed on a 7T Small Animal Scanner (Biospec 70/30, Bruker, Ettlingen, Germany). The animal was positioned in the magnetic field and, after initial scout acquisitions, the aortic bifurcation was identified. A T1w two-dimensional double inversion-recovery (DIR) black blood (BB) RARE sequence [37] (TR/TE/TI: 1000/10/350 ms, RARE factor 2, field of view (FOV) 12x12cm, acquired/reconstructed matrix size 384x384/512x512, 20 transversal adjoining slices with a thickness of 3 mm) was acquired with the bottom slice positioned at the aortic bifurcation. After visual inspection of these images, a single slice with the highest plaque burden was selected. DCE-MRI was performed at this location using a two-dimensional inversion-recovery black blood turbo spin echo sequence [20] (TR/TE/TI = 300/9.5/120 ms, field of view 12x12cm, acquired/reconstructed matrix size 192x192/384x384 with a thickness of 3 mm) with an acquisition time of 7.2 seconds per time frame. After one minute, 0.2 mmol/kg body weight Gadobutrol (Gadovist, Bayer HealtCare, Berlin, Germany) was injected intravenously through a marginal ear vein manually at a rate of 0.5 ml/sec, followed by a 5 ml saline chaser at the same rate. Imaging was continued for 120 time frames. After, contrast-enhanced T1w DIR BB imaging was performed again using the same pulse sequence parameters as listed above.

#### Ultrasound image review and data analysis

The anterior and posterior lumen-wall interface were identified manually for each M-line. A wall track system [38, 39] was used to obtain the diameter waveforms over the cardiac cycle. A temporal correlation window (26 ms, 10 sample points) was used for assessment of diameter waveforms with high temporal resolution. Absolute distension of the lumen was calculated as the difference between systolic maximal diameter and preceding diastolic minimal diameter, while relative distension was determined as the absolute distension divided by the diastolic minimal diameter.

#### MR image review and data analysis

Lumen and outer vessel wall contours were drawn using dedicated vessel wall analysis software (VesselMASS, Leiden University Medical Center, Leiden, The Netherlands). Mean plaque burden was quantified on contrast-enhanced T1w BB MR images. From these, contours were transferred to the DCE MR images. Inner contours were adjusted to prevent partial volume effects at the plaque-lumen border.

The DCE-MRI signal intensity time curves (relative to baseline values before contrast injection) of the atherosclerotic vessel wall were quantified semi-quantitatively (area-under-the-curve, AUC) and quantitatively (K^trans^, indicative for plaque microvasculature flow, density, and permeability) with pharmacokinetic modeling using a reference region model [40]. With this method, the arterial input function is not required, allowing quantitative analysis of BB DCE-MRI acquisitions. The AUC was calculated by numeric integration. In line with previous research [20], integration is performed from 0–7 minutes after contrast injection. For the quantitative analysis, literature values for the blood perfusion-vessel permeability product (K^trans^) and the extravascular–extracellular volume fraction (v_e_) [41] for the reference region (skeletal muscle) were used.

#### Histological and immunohistochemistry analysis

After euthanasia, the abdominal aorta was dissected from the aortic bifurcation to ~7 centimeters proximally. Specimens were fixated using 1% paraformaldehyde for 24 hours, cut into 5-mm-thick tissue slices, processed and embedded in paraffin and sectioned (4-μm sections). An Elastica von Gieson (EvG) staining was used to visualize the inner and outer elastic laminae and the lipid core. Presence of cap fissures was examined using a haematoxylin eosin stain. Immunohistological staining for rabbit macrophages and microvessels was performed using a primary antibody against monocytes/macrophages (clone RAM11; Dako North America, Carpinteria, California) and a primary antibody against CD31 (clone JC70A, Dako), respectively. RAM11 and CD31 slides were counterstained with hematoxylin. Matching of histologic sections with MRI slices was performed based on their longitudinal position relative to the aortic bifurcation, taking into account an ex vivo shrinking of approximately 15%. Digital images from slides matched to the DCE-MRI slice were analyzed with morphometric analysis software. EvG and RAM11 images were analyzed using QWin V3 (Leica, Cambridge, Great Britain) and CD31 images using Ventana Image Viewer (Ventana, Tucson, Arizona). Median intima size and relative lipid rich necrotic core size (relative to the intima size) were determined on the EvG stained sections. Lipid rich necrotic core was identified by the area of necrotic material in which residual cholesterol crystals were seen. The relative macrophage content was determined as the amount of RAM11 positive area related to the total plaque area. Presence of macrophages in the proximity (approximately 50 μm) of the lumen was scored semi-quantitatively on a three point scale ranging from no luminal macrophages (0) to many luminal macrophages (2). Microvessel density was determined as the number of microvessels per mm^2^ for the entire vessel wall. All histological analyses were performed blinded to the other study results and animal characteristics.

#### Statistical analysis

The differences between study parameters of the two groups were studied using an independent Mann-Whitney U Test. Study parameters were divided into cardiovascular parameters (conscious and anesthetized heart rate, and systolic and diastolic BP), plaque burden parameters (mean vessel wall area on MR images and mean histological intimal area), plaque biomechanical parameters (absolute and relative vessel wall distension), and plaque vulnerability markers (plaque enhancement from DCE-MRI determined using AUC and K^trans^, and relative lipid-rich necrotic core size, microvessel density, and macrophage content from histology). Relationships between macrophage content and DCE-MRI parameters (AUC and K^trans^), and between microvessel density, AUC, and K^trans^ were assessed using a Spearman’s correlation coefficient. All results were considered significant for a p-value < 0.05.

## Results

The number of animals in which successful experiments were performed is listed in Table 1. During the study, two rabbits (one non-treated and one Ivabradine-treated) died due to complications during the balloon denudation surgery. One Ivabradine-treated animal died during the study period due to icterus, caused by the cholesterol-enriched diet (diagnosed by a veterinary doctor at autopsy). For one non-treated animal, contrast injection failed. Therefore, this animal was excluded from MRI and histological analysis. For one Ivabradine-treated animal, BP measurements failed.

**Table 1:** Number of successfully performed procedures in the present study.

Experiment	Non-treated	Ivabradine-treated
Included animals	10	9
Conscious HR	9	7
Blood Pressure	9	6
Ultrasound Imaging	9	7
Magnetic Resonance Imaging	8	7
Histology	8	7

### Heart rate and blood pressure

**Fig 2:**
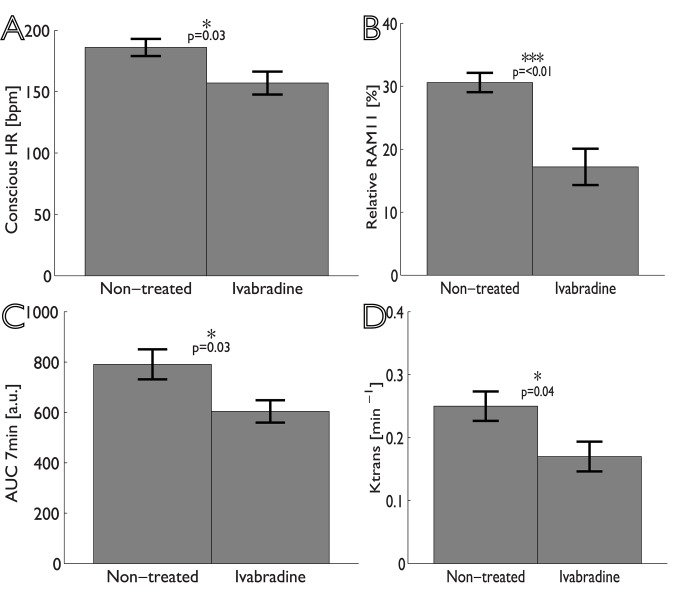
Bar graphs showing the differences between non-treated and Ivabradine-treated animals (mean ± standard error). Panel A shows the conscious heart rate; panel B relative RAM11 positive area in histological sections; panel C and D show the area-under-the-curve (AUC) and K^trans^ determined from analysis of DCE-MR images.

As shown in Table 2, HR for Ivabradine-treated animals was significantly lower than for non-treated animals, both under conscious (Fig 2A) conditions (mean reduction 16%, p = 0.026), and under anesthesia (mean reduction 15%, p = 0.031). No significant differences were found for the systolic and diastolic BP measured under anesthesia between groups.

**Table 2:** Study parameters for the present study. Parameters are reported as mean values ± standard error. Number of successful experiments that were performed is indicated (^§^n = 9, ^ll^n = 8, ^¶^n = 7, ^#^n = 6). AUC 7 min and K^trans^ represent DCE-MRI parameters of the plaque microvasculature. The percentage of RAM11 is a measure of the plaque macrophage content. Used abbreviations: heart rate (HR); blood pressure (BP); Elastica von Gieson (EvG); area-under-the-curve (AUC); ^*^p<0.05.

Category	Parameter	Non-treated	Ivabradine-treated	P-value
Cardiovascular	Conscious HR [bpm]	186±7.0^§^	157±9.3^¶^	0.03^*^
	Anesthesia HR [bpm]	164±5.9^§^	140±6.4^¶^	0.03^*^
	Systolic BP [mmHg]	63±3.4^§^	71±7.9^#^	0.27
	Diastolic BP [mmHg]	45±3.4^§^	53±6.8^#^	0.33
Plaque burden	MR plaque size [mm^2^]	11.0±0.9^ll^	10.8±1.1^¶^	0.54
	EvG intima size [mm^2^]	6.80±1.03^ll^	6.61±1.19^¶^	0.91
Biomechanics	Absolute distension [mm]	0.066±0.017^§^	0.064±0.018^¶^	0.84
	Relative distension [%]	1.9±0.4^§^	1.9±0.3^¶^	0.84
Plaque vulnerability	AUC 7min [a.u.]	791±60^ll^	604±44^¶^	0.03^*^
	K^trans^ [min^-1^]	0.25±0.02^ll^	0.17±0.02^¶^	0.04^*^
	Microvessel density [mm^-2^]	12.9±1.8^ll^	9.8±1.8^¶^	0.40
	Relative lipid core size [%]	1.7±0.4^ll^	1.5±0.3^¶^	0.66
	Relative RAM11 [%]	30.6±1.5^ll^	17.2±2.9^¶^	<0.01^‡^

### Plaque biomechanics

No differences were found in beat-to-beat plaque biomechanical parameters between the two groups (Table 2). However, due to the HR reduction, the frequency that the aorta is exposed to the pulsatile BP is decreased for Ivabradine-treated animals.

### Plaque features

**Fig 3:**
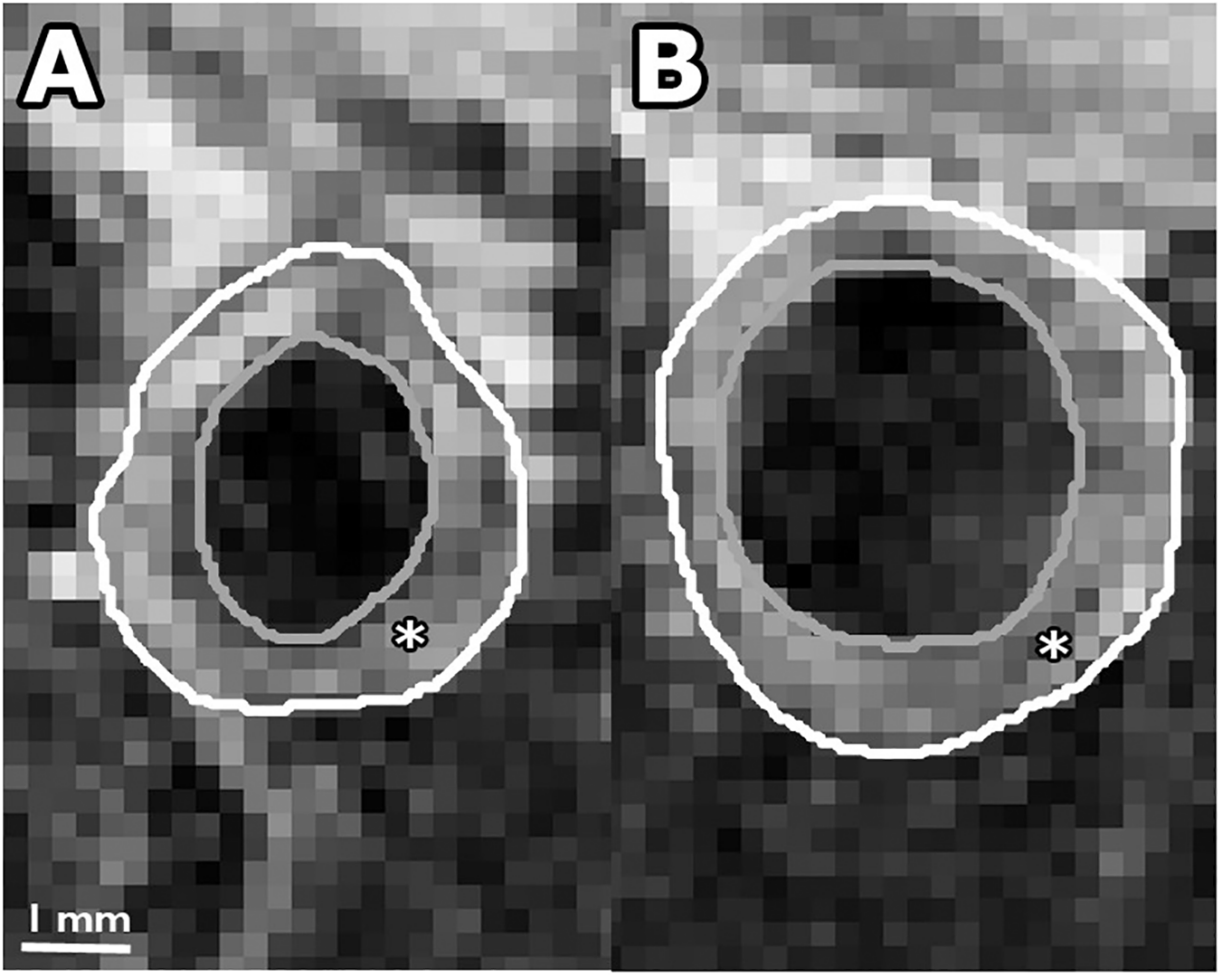
Contrast-enhanced T1w BB MR image of atherosclerotic plaque for a non-treated rabbit (panel A) and Ivabradine treated rabbit (panel B). In the panels, the outer vessel wall is indicated by the white line and the lumen by the gray line. The atherosclerotic plaque is indicated by the asterisk (*).

**Fig 4:**
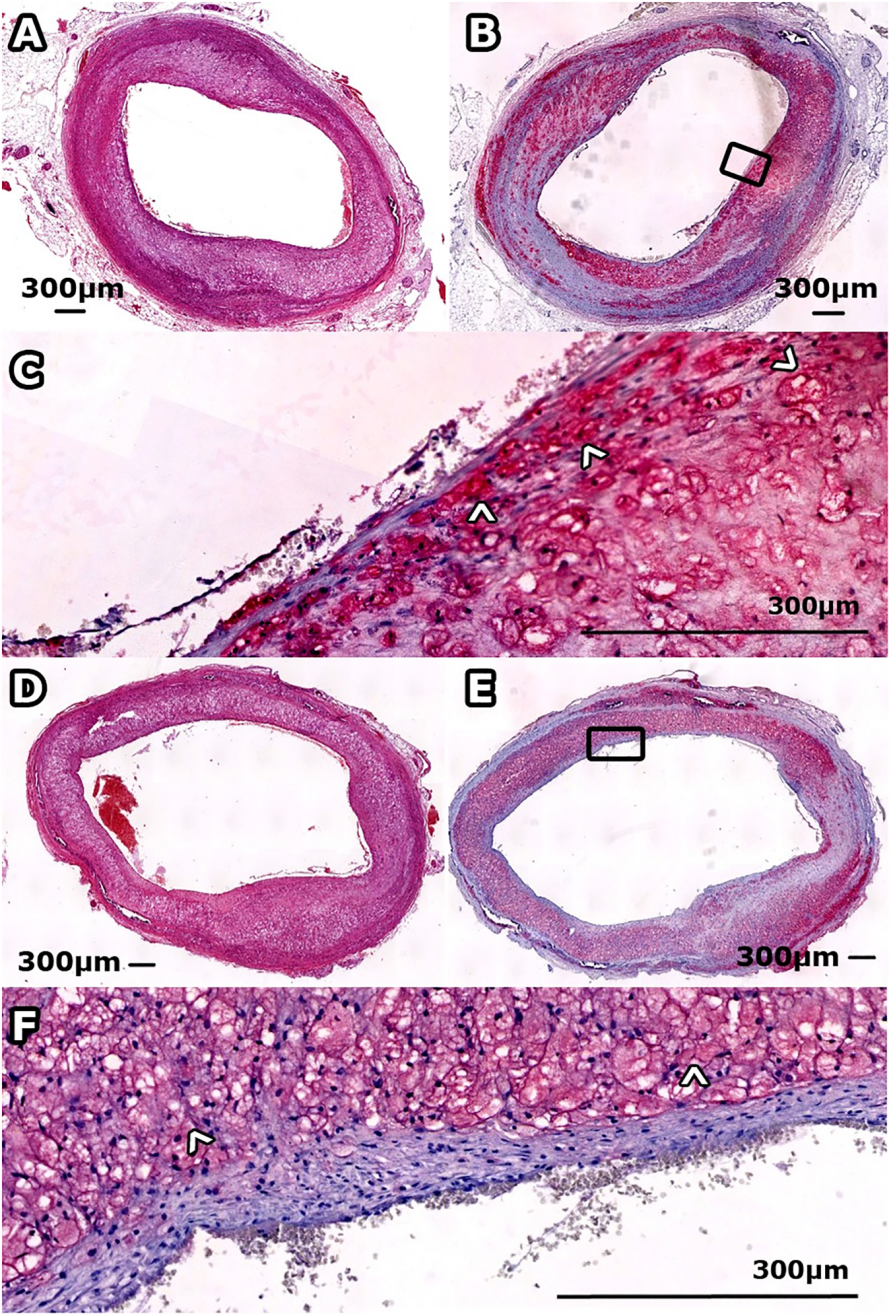
Histological sections of atherosclerotic plaque for a non-treated (panels A-C) and Ivabradine treated (panels D-F) rabbit. Panel A and D show HE sections and panel B and E a slide stained for macrophages using RAM11 antibody (red), with magnifications in panel C and F.

**Fig 5:**
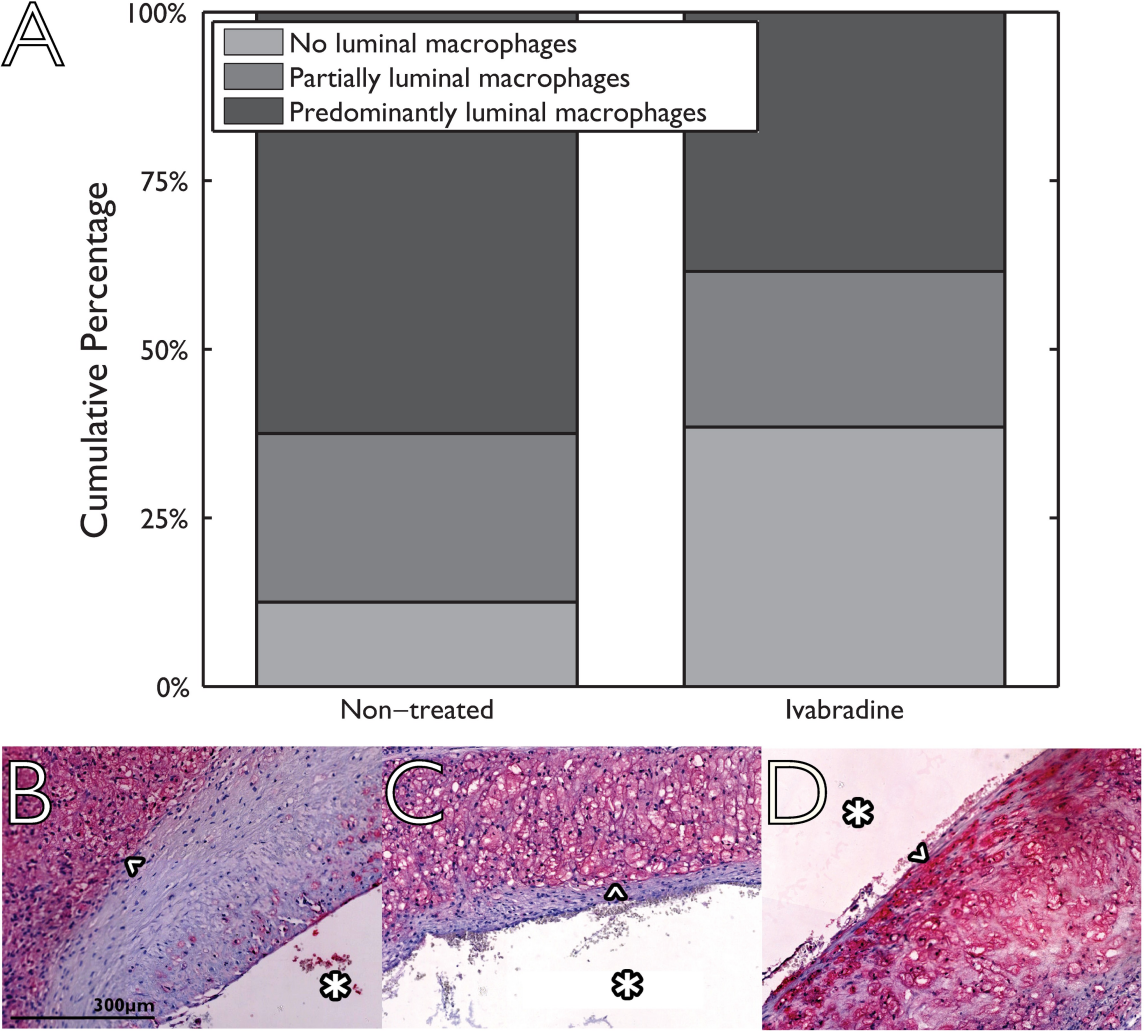
Presence of macrophages in the proximity of the vascular lumen using a semi-quantitative three point scale for non-treated (eight animals, two sections per animal) and Ivabradine-treated animals (seven animals, 2 sections per animal). Histological sections are divided into three categories ranging from 0 (no luminal macrophages; example image shown in panel B) via 1 (partially luminal macrophages; example in panel C) to 2 (predominantly luminal macrophages; example in panel D). Macrophages in the histological images are indicated by an arrow head (^). The displayed scale bar is applicable for all histological images and the luminal side is indicated by an asterisk (*) in all histological images.

**Fig 6:**
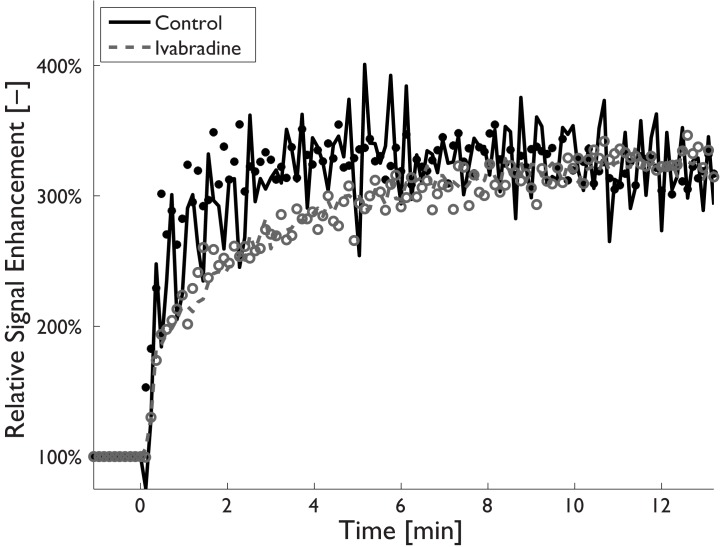
Dynamic contrast-enhanced MRI signal-intensity-time curves for a non-treated (black filled circles) and Ivabradine-treated animal (red open circles) showing the relative signal enhancement in the atherosclerotic vessel wall after contrast injection. Pharmacokinetic modelling curves are shown as black solid lines (non-treated animals) and gray dotted lines (Ivabradine-treated animals). Enhancement curves for Ivabradine-treated animals show a delayed enhancement compared to non-treated animals, which is indicative for a decrease in plaque microvasculature.

Examples of MR images and histological sections (HE and RAM11 antibody against macrophages) for non-treated and Ivabradine-treated animals are shown in Figs 3 and 4, respectively. No significant differences were found for plaque burden related parameters (Table 2), as determined using MRI (mean entire vessel wall area) and histology (mean intima area). Also, no differences were found for the relative lipid core size between the two groups. The relative RAM11 positive area (macrophages) was decreased from 30.6±4.3% for non-treated animals to 17.2±7.6% for Ivabradine-treated animals (p<0.001). Plaques of the Ivabradine-treated group showed less macrophages in proximity of the vessel lumen as compared to non-treated animals (Fig 5). Presence of cap fissures was not observed in the specimens. Analysis of the DCE-MRI data (Fig 6) revealed a reduction in vessel wall enhancement, both semi-quantitatively (24% reduction in the AUC after seven minutes, p = 0.029) and quantitatively (32% reduction for K^trans^, p = 0.037) for rabbits treated with Ivabradine, indicative for a decrease in plaque microvasculature flow, density, and/or leakiness. A strong positive correlation between AUC and K^trans^ (Spearman’s ρ = 0.70, p = 0.004) was observed. No difference in microvessel density in histology from the entire vessel wall was found between non-treated (12.9±1.8 mm^-2^) and Ivabradine-treated (9.8±1.8 mm^-2^) animals (p = 0.397). Microvessel density in histology correlated with AUC (Spearman’s ρ = 0.53, p = 0.041), but not with K^trans^ (Spearman’s ρ = 0.17, p = 0.550). A modest, non-significant, correlation between the relative RAM11 area and the signal enhancement on DCE-MRI with a Spearman’s ρ of 0.46 (p = 0.081) for the AUC after 7 minutes and a Spearman’s ρ of 0.49 (p = 0.062) for K^trans^ was found.

## Discussion

The major novelty of the present study is the use of sophisticated noninvasive imaging techniques in combination with histology to study the effect of the HR lowering agent Ivabradine on atherosclerotic plaques. This allowed us to demonstrate that HR lowering treatment was associated with a reduction in macrophage content and changes in contrast enhancement of rabbit plaques, indicative for less inflammation and a decrease in plaque microvasculature flow or leakiness, respectively. In contrast, no differences in plaque burden, microvessel density, and vessel wall distension were observed between treated and non-treated rabbits.

DCE-MRI enables to study not only plaque microvascular density, but also microvascular leakiness. Our qualitative as well as quantitative DCE-MRI parameters, indicative for microvascular density, flow, and leakiness, showed a reduction after Ivabradine treatment, while the microvessel density on histology showed no difference between non-treated and Ivabradine-treated animals. Together, these results suggest a reduction of the plaque microvasculature leakiness.

With a decreased HR, the vascular wall has a lower exposure to oscillatory tensile and wall shear stresses [42, 43], decreasing the frequency of the biomechanical load. A reduction in shear and tensile stress is thought to reduce endothelial damage, which leads to a decreased endothelial permeability to inflammatory mediators [42, 43], in line with a decrease in microvascular leakiness and a reduction in macrophage content in the treatment group. Alternatively, the treatment may have reduced microvessel rupture, since it was previously suggested that pressure gradients within the plaque that occur during every heart beat lead to microvessel rupture [44]. In line with a decrease in endothelial permeability, Ivabradine-treated animals showed less luminal macrophages (Fig 3), while in both animal groups no cap fissures were found.

In the present study, HR reduction was induced during the entire study period. Future studies with a longitudinal design in which HR reduction is performed after atherosclerosis induction in animal models are warranted to investigate whether HR reduction reduces plaque vulnerability or mitigates the formation of plaque vulnerability. Future studies should also investigate the effect of Ivabradine and more commonly applied HR reducing agents, i. e. beta blockers, on plaque vulnerability in patient studies.

For the current study, a non-significant, albeit modest, correlation between RAM11 positive area (relative macrophage content) and DCE-MRI parameters was found. These results are in line with previous studies [20, 23, 28–30], where a weak to modest correlation between plaque inflammation and microvasculature was reported. The present study supports the concept that there is a complex interplay between macrophages and plaque microvasculature [28]. Macrophages have a high metabolic rate, which can lead to plaque hypoxia, which is known to stimulate angiogenesis [45]. The newly formed, potentially immature angiogenic microvessels may then allow additional inflammatory cells to enter the plaque tissue.

The extent of HR reduction in our study is in line with a previous study in ApoE^-/-^ mice [35]. In contrast to this previous study, in our rabbit model administration of Ivabradine did not lead to smaller plaques on MRI and histology in the treatment group. The differences in plaque burden between our study and previous findings might be related to differences between the two animal models [46–48]. In the previous study [35], administration of Ivabradine did lead to reduced endothelial dysfunction and exerted potent anti-oxidative effects in ApoE^-/-^ mice. Following these results, further in vitro investigations were performed to investigate if Ivabradine exerted direct effects on the endothelium. These investigations revealed no differences of the vasodilatation of vessel segments or protein expression of cultured bovine aortic endothelial cells, suggesting that direct anti-oxidative effects of Ivabradine are not involved in the reduction of endothelial dysfunction.

Microvessel density in the non-treated group was higher than our findings in a previous study [36]. The higher microvessel density may be related to the extended cholesterol-enriched diet period of the present study.

A number of mechanisms of Ivabradine next to a reduction of the HR were observed that improve myocardial structure and function, including anti-inflammatory effects, myocardial infarct size reduction, a reduction of the formation of reactive oxygen species, increased coronary flow reserve, and an increased perfusion of the coronaries [49]. While previous research [50] did show improvements in clinical outcome (cardiovascular death or hospital admission for worsening of heart failure) in patients with heart failure after HR reduction with Ivabradine, a recent study in patients with stable coronary disease without clinical heart failure [51], showed that additional treatment with Ivabradine did not lead to a reduction in cardiovascular death or nonfatal myocardial infarctions. Therefore, it is of great interest to further investigate which patient groups may and may not benefit from HR lowering treatment with Ivabradine [52, 53].

A number of mechanisms of Ivabradine next to a reduction of the HR were observed that improve myocardial structure and function, including anti-inflammatory effects, myocardial infarct size reduction, a reduction of the formation of reactive oxygen species, increased coronary flow reserve, and an increased perfusion of the coronaries [49]. While previous research [50] did show improvements in clinical outcome (cardiovascular death or hospital admission for worsening of heart failure) in patients with heart failure after HR reduction with Ivabradine, a recent study in patients with stable coronary disease without clinical heart failure [51], showed that additional treatment with Ivabradine did not lead to a reduction in cardiovascular death or nonfatal myocardial infarctions. Therefore, it is of great interest to further investigate which patient groups may and may not benefit from HR lowering treatment with Ivabradine [52, 53].In the present study, the animals’ HR was measured once before the MR and US examinations performed after 14 weeks. Continuous monitoring of the HR during the study period using telemetric devices was not performed due to logistical reasons. Compared to previous research [20–24] within the present study, analysis of the DCE-MRI data was extended with quantitative analysis using a reference region method. The reference region method does not require a vascular input function and thus allows quantitative analysis of black blood (BB) DCE-MRI data. It is able to account for inter-subject variations due to contrast bolus differences. Use of BB images allows for more reliable determination of the plaque microvasculature near the lumen, which is important in the small-sized aortic rabbit plaques. Our study shows a strong, significant correlation between the semi-quantitative AUC and quantitative K^trans^ parameter.

Our study has a number of limitations. First, we have chosen Ivabradine as a heart-rate reducing agent to investigate the specific effect of heart-rate reduction, while not affecting cardiac contractility or blood pressure (BP) [33, 34]. However, a recent study on mouse ventricular myocytes has shown that Ivabradine blocks the IhERG1 potassium current, which appears to contribute to a prolonged cardiac repolarization [54]. Therefore, we cannot exclude that the present findings have been influenced by other actions of Ivabradine. Second, DCE-MR imaging was performed on a single axial slice. Previous research [20–24] has shown that this technique is able to quantify the plaque microvasculature and measure the effect of therapeutic interventions. Recently, a 3D acquisition techniques to quantify plaque microvasculature in rabbits have been introduced [55]. This 3D technique provides increased spatial coverage as compared to the 2D technique, although at the expense of temporal resolution.Finally, from previous studies it is known that the cardiovascular hemodynamics are affected by the administration of general anesthesia, with the effect differing between species and strains and the type of anesthetics and administered dose [56–58]. Isoflurane is known to have vasodilative properties, which may lead to hypotension [58]. General anesthesia by administration of a combination of ketamine and xylazine should potentially not lead to hypotension and may be more suited to perform measurements under physiological conditions that resemble normal conditions more closely. However, the increased wake-up and recovery period may increase complications [58].

### Conclusion

Our study suggests that a lower HR may lead to reduced plaque microvessel leakiness and reduced plaque inflammation in a model of atherosclerotic rabbits. Increased plaque inflammation and plaque microvasculature leakiness have been suggested as key features of plaque vulnerability. The present study suggests that exposure of the atherosclerotic vessel wall to biomechanical load at a higher repetition frequency contributes to plaque vulnerability. HR reduction may be a potential target for plaque stabilization. Whether these preclinical findings can be translated into clinical practice remains to be determined.

## Supporting information

S1 File Data underlying the study findings. (XLSX)
